# Exposure to Movie Reckless Driving in Early Adolescence Predicts Reckless, but Not Inattentive Driving

**DOI:** 10.1371/journal.pone.0113927

**Published:** 2014-12-10

**Authors:** Evelien Kostermans, Mike Stoolmiller, Rebecca N. H. de Leeuw, Rutger C. M. E. Engels, James D. Sargent

**Affiliations:** 1 Behavioral Science Institute, Radboud University Nijmegen, Nijmegen, the Netherlands; 2 Donders Institute for Brain, Cognition and Behavior, Radboud University Nijmegen, Nijmegen, the Netherlands; 3 College of Education, University of Oregon, Eugene, Oregon, United States of America; 4 Department of Pediatrics, Geisel School of Medicine at Dartmouth, Lebanon, New Hampshire, United States of America; Nathan Kline Institute and New York University School of Medicine, United States of America

## Abstract

**Objective:**

We examine the association between exposure to depictions of reckless driving in movies and unsafe driving, modeling inattentive and reckless driving as separate outcomes.

**Methods:**

Data were obtained by telephone from 1,630 US adolescents aged 10 to 14 years at baseline who were drivers at a survey 6 years later. Exposure to movie reckless driving was measured based on movies seen from a randomly selected list of 50 movie titles that had been content coded for reckless driving among characters. Associations were tested with inattentive and reckless driving behaviors in the subsequent survey–controlling for baseline age, sex, socioeconomic status, parental education, school performance, extracurricular activities, daily television and video/computer game exposure, number of movies watched per week, self-regulation and sensation seeking.

**Results:**

Exposure to movie reckless driving was common, with approximately 10% of movie characters having driven recklessly. Confirmatory factor analysis revealed a significant distinction between items tapping reckless and inattentive driving at the 6^th^ wave. Age and exposure to movie reckless driving at baseline were directly associated with wave-6 reckless (but not inattentive) driving. Additionally, growth in sensation seeking mediated a prospective relation between the total number of movies watched per week at baseline and reckless driving, independent of exposure to movie reckless driving. Males and high sensation seekers reported lower seatbelt usage and more reckless driving, whereas lower self-regulation predicted inattentive driving.

**Discussion:**

In this study, exposure to movie reckless driving during early adolescence predicted adolescents’ reckless driving, suggesting a direct modeling effect. Other aspects of movies were also associated with reckless driving, with that association mediated through growth in sensation seeking. Predictors of reckless driving were different from predictors of inattentive driving, with lower self-regulation associated with the latter outcome. Making a clear distinction between interventions for reckless or inattentive driving seems crucial for accident prevention.

## Introduction

In the USA, road traffic crashes are the leading cause of death for 15-to-20-year-olds; in 2008, 2,739 drivers in this age group were killed and an additional 228,00 were injured in motor vehicle crashes [1]. Compared to any other group, the risk to be in a fatal crash is substantially higher for 16- to 19-year-olds [2]. Among youth, the high rate of traffic accidents result from inexperience [3], [4] and a higher tendency to drive recklessly (e.g., speeding, following too closely) [5], [6]. In fact, reckless driving accounted for approximately 51% of the total economic crash costs ($230.6 billion) of all 16.4 million U.S. motor vehicle collisions in 2000 [7].

To reduce the number of (fatal) traffic accidents among adolescents, expansion of the current knowledge of risk factors is crucial. Risk factors for reckless driving known so far include male gender, younger age, and higher sensation seeking tendencies [8]–[13].

In addition to individual characteristics, positive feelings towards driving have been associated with reckless driving tendencies [8]. These positive driving-related emotions are enhanced by the marketing of automobiles that focus on the pleasure and fun of driving [14]. A meta-analytic review of Fischer and colleagues [15] demonstrated positive associations between risk-glorifying media and risk taking behaviors for a number of health related risky behaviors such as smoking, alcohol consumption and reckless driving, both on the short and long term and across different research methods (experimental, correlational, longitudinal). Effects were strongest for active (video games) versus passive media consumption (advertisement, movies, music) and when there was a match between media content and type of risk taking. With regard to reckless driving, nearly all studies focused on active media consumption (racing games), and showed that playing racing games is associated with risk taking in simulated traffic situations [16]–[18] and self–reported reckless driving [19], [20]. One study [17] demonstrated a short-term positive link between watching risk glorifying movie scenes and risky driving on a racing game (*Need for Speed,* EA Games). Importantly, these experimental studies demonstrated a link from exposure to risk –positive cognitions, emotions and risk-taking behaviors [21].

Fischer and colleagues [21] proposed a theoretical framework that extends socio-cognitive models of learning [22] and the more recent General Learning Model [23], and explains elevated levels of risk taking in relation to media exposure not only through priming effects of risk-positive cognitions and emotions, but also through changes in the self-concept, due to (1) situational cues in the media that risk taking is rewarding instead of potentially dangerous, (2) through habitation processes and changes in risk-related social norms, and (3) through identification processes that are stronger in active vs. passive media consumption.

In addition to active media consumption (video games), passive media such as movies, television shows [24] and automobile commercials [25] frequently contain portrayals of reckless driving and may even reach a broader public than videogames, which are played more commonly by males [26]. To illustrate the potential impact of passive media consumption, Vitaglione showed that after live broadcasting of the extremely popular and widely viewed National Association for Stock Car Racing (NASCAR) events that display dangerous and risky automobile racing, the number of driving accidents and traffic injuries caused by aggressive driving increased, but only after 5 days following the broadcast [24]. Vitaglione suggested that this delayed increase could be due to accumulating priming effects during the consecutive days after the broadcast and only after a certain threshold had been passed, influenced dangerous driving behaviors.

Few studies have focused on the long-term relation between exposure to passive forms of risk-glorifying media consumption and driving behavior. Buellens, Roe, and Van den Bulck [27], [28] demonstrated indirect long-term associations between viewing action movies or music videos and reckless driving attitudes and intentions. However, these studies did not control for socioeconomic status or parenting style and failed to formally link exposure with behavior by testing for mediation processes between attitudes and reckless driving. Additionally, reckless driving has not been examined in relation to driving-related passive media that occurs well prior to driving debut. Therefore, the present study examines the long-term role of reckless driving-related movie exposure, while controlling for important background variables and also testing for mediation processes.

Teenage drivers are not only more willing to take risks in traffic, but are also more likely to engage in distracted and inattentive driving [29]–[31] Therefore, we also determine whether an existing measure of unsafe driving may be used to model reckless and inattentive driving separately, since both may contribute to traffic accidents by different mechanisms [6], [8].

To differentiate the processes underlying unsafe driving behavior in this study, a distinction was made between inattentive and reckless driving outcomes. Inattentive driving is not volitional—it involves a failure to notice and respond appropriately to a key element in the traffic environment due to distractions inside or outside the car [32]. Inattentive driving was therefore hypothesized to be associated with lower levels of self-regulation, of which a key component is the ability to maintain focused attention [33]. On the other hand, reckless driving is intentional—involving, for example, the decision to speed or weave in and out of traffic. These types of decisions were hypothesized to be associated with higher levels of sensation seeking [34]. In line with the theoretical framework on the impact of risk-glorifying media exposure on risk taking inclinations of Fischer and colleagues [21], we hypothesized that higher passive exposure to risky driving movie depictions during early adolescence will predict reckless but not inattentive driving. Similar to previous research [35], we expected that increases in sensation seeking will mediate the relation between watching reckless driving and engaging in the behavior.

## Methods

### Participants and procedure

We surveyed 6,522 U.S. adolescents between the age of 10 to 14 years by telephone in 2003 and followed them forward in 5 additional survey waves [36]. Adolescents were recruited using random-digit dialing and surveys conducted by trained interviewers who using computer-assisted telephone interviews. All aspects of the study were approved by Dartmouth IRB. Basic demographics such as socioeconomic status, race/ethnicity and census region for the baseline sample mirror those for U.S. adolescents between 10 and 14 years of age [37]. Three consecutive follow-up measurements (waves 2–4) were conducted at 8-month intervals, and about 2 years separated waves 4, 5 and 6, with sample retention at wave 4 being 4,574 (70%), 3,055 (47%) at wave 5, and 2,322 (36%) at wave 6. To minimize differences in driving experience, we selected as the analytic sample those teens (N = 1,647) who reported driving experience at both waves 5 (*M* = 16.08, *SD* = 1.27) and 6 (*M* = 18.22, *SD* = 1.31). Of these teens, 17 were dropped from the analysis due to missing wave 1 data, so modeling results are based on 1,630 teens.

#### Outcome: Unsafe driving behavior

At wave 6, all drivers were asked about their unsafe driving practices using 9 dichotomous (yes/no) items drawn from The National Survey of Speeding and Other Unsafe Driving Actions, conducted by the National Traffic Safety Administration in 1998 [38] Inspection of the items suggested that some measured inattentive driving (e.g., failed to yield) and others reckless driving (e.g., weaved in and out of traffic), [29], [39]. Further evidence of a distinction is included in the analysis section.

#### Exposure: Movie reckless driving exposure

Adolescents’ exposure to movie reckless driving was assessed by using the previously validated Beach method [34]. Top box office hits released in the 5 years prior to the baseline survey were content analyzed for reckless driving, measured by enumerating all characters in each movie and assessing which frequently engaged in “fast, careless” driving. Krippendorf alpha for content coding of character reckless driving on a 10% sample of double-coded movies (1,868 major characters) was 0.99. Adolescents were asked whether they had seen each movie title on a unique list of 50, randomly selected from the larger pool. Based on movies the adolescent had seen, movie reckless driving exposure was calculated as the proportion of the characters they had seen that had engaged in reckless driving and trimmed at the 95^th^ percentile to limit high outlier influence [40].

#### Mediators

Sensation seeking was measured using a previously validated scale (Crohnbach’s alpha  = 0.60), [41] that included items identified by Zuckerman [34], and the Arnett Inventory of Sensation Seeking (e.g., “ I like to do dangerous things,”), [42]. Self-regulation was measured with a four-items based on the Kendall Wilcox scale (e.g., “I am good at waiting my turn”) [43].

#### Covariates

The analysis controlled for variables that could be associated with exposure to reckless driving in movies and also with reckless driving. Covariates including sex, age, socioeconomic status, and parental education, self-reported school performance, extracurricular activities, daily television exposure, number of movies watched per week, and the number of hours a day spent playing video or computer games. Socio-economic status (SES) was determined by combining the variables parent education and household income into a standardized SES score. Parenting style was assessed by the Authoritative Parenting Index [44]. Rebelliousness was measured using a composite 4-point Likert-type scale of six items assessing tendency toward antisocial behavior [45],[46] (e.g., ‘‘I like to break the rules,’’, ‘‘I argue a lot with other kids’’). All questions capturing the covariates are presented in S1 Table.

### Data analysis

First, we assessed whether the Unsafe Driving Behavior outcome could be split into two domains using factor analysis. Based on the literature and conceptual interpretation, we made a distinction between intentional unsafe driving behaviors such as speeding or tailgating [5], [6] and driving behaviors that are more likely to result from inattentive or distracted driving, such as failing to yield right of way [47] or driving through stop signs [48]. The reckless factor was defined a priori by the items of speeding, tailgating, weaving, passing on a double yellow line, speeding through a yellow light and not wearing a seat belt. The inattentive factor was defined a priori by the items of failing to yield, ignoring a stop sign and running a red light. It should be noted that the items “speeding through a yellow light” and “running a red light” are obviously closely related, as deliberately speeding through a yellow light increases the chances of unintentionally running a red-light as well. In so-called “dilemma zones”, drivers are too late to stop their vehicle in time but are also too late to enter the intersection before the light turns red [49]–[50]. Whereas the first item unambiguously captures deliberate behavior, the latter item is more ambiguous. Previous research [51]–[53] suggests that red-light running can be categorized both as “intentional”, referring to deliberate red-light running to avoid delays or out of frustration, or “unintentional”, referring to drivers that do not see the red light because of distractions, inattentive driving, or obstructed sight (e.g., sun glare, vegetation). In the majority of cases, red-light running appears to be unintentional [51]. Therefore, we choose to regard the item “speeding through a yellow light” as intentional, and “running a red light” as primarily unintentional. We also hypothesized that a model with 2 correlated factors (one for reckless driving and one for inattentive driving) would fit better than a 1 factor model.

If this were true, the two latent factors would serve as the ultimate outcomes in a structural equation model that also included sensation seeking measured at wave 4 as a potential mediator of the wave 1 risk factors of movie reckless driving and sensation seeking and the wave 1 control variables. The structural equation model was specified as a standard, linear model with all latent variables assumed to be normally distributed. The unsafe driving items were connected to the wave 6 latent factors via probit or logistic regressions. Because the model included both linear and logistic or probit regressions simultaneously, estimation was carried out using numerical integration available in Mplus 7 [54]. After model fitting, we computed the following types of effects for wave 1 risk factors on the wave 6 unsafe driving items: direct, indirect through a w6 latent construct, and double indirect through both wave 4 sensation seeking and a wave 6 latent construct. For wave 4 sensation seeking, we computed direct and indirect effects through a wave 6 latent construct. All effect sizes were computed on the odds scale to provide a familiar metric. These computations were carried out using nonlinear constraints in Mplus, which uses the delta method to obtain standard errors. A priori hypotheses about direct and indirect effects were tested using a p value of.05 or less.

For parsimony, the model incorporated 2 broad sets of simplifying assumptions. First, except as noted in the introduction for sensation seeking, movie reckless driving and self-regulation, we assumed that the rest of the effects of the wave 1 and wave 4 risk factors and covariates would be the same on both latent factors. Second, we assumed that all wave 1 and wave 4 effects on the unsafe driving items were indirect through the two wave 6 latent factors. Both sets of assumptions were checked and relaxed if there was strong evidence (using a more stringent p value of.001 due to the number of tests involved) that they were inappropriate, and all models were estimated using robust maximum likelihood.

## Results

### Descriptive Statistics

S2 Table reports descriptive statistics for all covariates for the analytic sample (n = 1647) at baseline. Adolescents from each age group and household SES stratum were present. Few had little-to-no exposure to TV and movies. Approximately 10% of the characters in the 532 movies drove recklessly. Adolescents had seen a mean of 16 (*SD* = 7.26) of the 50 movies on their individualized list, through which they received a mean exposure proportion 0.09 (interquartile range, 0.06 to 0.12) for proportion of characters that drove recklessly.

Table 1 also gives response percentages on the separate items of reckless and inattentive driving. Adolescents reported a mean of 2.61 (*SD* = 1.43) of the 6 reckless driving items (interquartile range, 2 to 3) and a mean of.65 (*SD* = .86) of the 3 inattentive driving items (interquartile range, 0 to 1). Some, such as exceeding the speed limit, were present in a substantial majority of respondents, whereas some (e.g., passing on a double line) were present in only a small minority.

**Table 1:** Self reported unsafe driving behaviors at wave 6.

Think about your driving in the past year. Have you ever done any of the following while driving? Have you…(Response options were “yes” or “no”)	Percentages of all adolescents (*n* = 1647) that responded “Yes”	Percentages of boys (*n* = 856) that responded “Yes”	Percentages of girls (*n* = 791) that responded “Yes”
… exceeded the speed limit?^a^	84.1%	83.3%	85.0%
… tailgated?^a^	31.1%	31.9%	30.2%
… failed to yield?^b^	23.9%	25.8%	21.9%
… weaved in and out of traffic?^a^	27.7%	29.1%	26.2%
… ran one or more red traffic lights?^b^	21.7%	20.9%	22.5%
… ignored stop signs?^b^	19.7%	22.7%	16.4%
… passed on a double line?^a^	13.6%	16.2%	10.7%
… sped through a yellow light?^a^	75.3%	75.5%	75.1%
… driven without your seatbelt fastened?^a^	29.0%	34.6%	22.9%

a Refers to items that capture reckless driving.

b Refers to items that capture inattentive driving.

#### Factor analysis of unsafe driving

We conducted a confirmatory factor analysis (CFA) using a probit link function.

We used the Weighted Least Squares Mean Adjusted and Variance Adjusted (WLSMV) probit model primarily because the probit model output and diagnostics are richer than the corresponding ML logit model for dichotomous indicators. We replicated the final CFA model using both approaches because ultimately, we wanted to focus on odds ratios as effect sizes, which are only available using the logit model.

WLSMV probit results showed that the fit of the two dimensional model was reasonable (χ^2^ [*df* = 26, *n* = 1,647] = 119.25, CFI  = .956, RMSEA  = .047). Modification indices for covariances among measurement errors suggested that allowing the items “run one or more red lights” and “speed through a yellow light” to correlate would substantially improve the model fit (and it also made sense conceptually that these two items were related). This additional path improved the fit (*χ^2^* [*df* = 25, *n* = 1,647] = 90.47, CFI  = .969, RMSEA  = .040). Although that model still had several large modification indices of around 20, none made conceptual sense and adding the largest made the model more difficult to interpret so we stopped considering additional changes to the hypothesized model. To test the fit of a one-factor solution as compared to the proposed two-factor solution, we forced the correlation between the two factors to be 1.0. Results show that the fit of the model deteriorated as compared to the two-factor solution; (*χ^2^* [*df* = 26, *n* = 1,647] = 154.54, CFI  = .939, RMSEA  = .055). A nested chi-square test comparing the one vs. two factor model indicated that the 2 factor model (correlation  = .69, 95% CI  = .61,.77) fit significantly better than a single factor model (nested *χ^2^* [*df* = 1, *n* = 1,647] = 54.34, *p*<.001).

To further examine the concurrent validity of the two risky driving constructs, we regressed wave 6 subject reports about being involved in a crash or being pulled over on the 2 latent constructs simultaneously. In the ML logit model, reckless driving significantly predicted both crashing (85% increase in odds) and being pulled over (224% increase in odds), net of inattentive driving but inattentive driving predicted neither, net of reckless driving. Results were similar in the corresponding WLSMV probit models.

### Structural Equation Model

**Figure 1:**
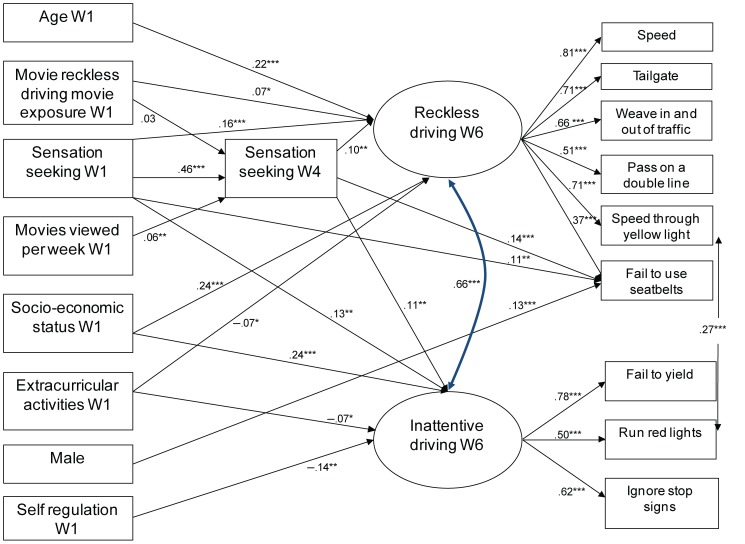
Path diagram of structural equation model including direct and indirect effects. Numbers on pathways represent standardized effects; *.01<*p*< = 0.05, **.001<*p*< = 0.01, ***p< = 0.001.

The most important direct and indirect effects discussed below are highlighted in Fig. 1.

#### Direct effects model

Simple, bivariate correlations between wave 1 predictors and wave 6 outcomes are provided in S3 Table. All of the direct effect models were estimated as WLSMV probit models. To set up the first model, we forced the latent variables reckless and inattentive driving to be perfectly correlated; we imposed equality constraints so that each wave 1 predictor had the same effect on both latent constructs; we specified all wave 1 predictor effects on the wave 6 indicators as indirect through the latent constructs (no specific direct effects); we included the correlated error term mentioned previously between “run one or more red lights” and “speed through a yellow light” so results would correspond to the measurement model discussed above. The fit of this baseline model, model 1, was marginal: χ^2^ [*df* = 146, *n* = 1,630] = 317.53, CFI  = .910, RMSEA  = .027. Perhaps more importantly, the effect of movie reckless driving on the single latent variable was not significant.

In the second step, we freely estimated the correlation between the two latent variables, which substantially improved the fit of model (χ^2^ [*df* = 145, *n* = 1,630] = 272.78, CFI  = .933, RMSEA  = .023) similar to results discussed above for the CFA measurement model. The nested chi-square was 37.72, *df* = 1, *p*<.001. The latent variable correlation was.68 (95% CI  = .60,.77).

In the third step, we conducted a priori tests concerning differential relations of w1 predictors with both latent constructs using a *p* value of.05. More specifically, the effect of the wave 1 predictor on reckless driving had to be significantly different at *p*<.05 than the corresponding effect on inattentive driving. Following the a priori tests, we considered relaxing the equality constraints on the relations of other wave 1 predictors with both latent constructs, starting with the predictor that resulted in the greatest improvement of fit provided the modification index indicated the improvement was significant at a more stringent *p* level of.001 or less. We stopped relaxing equality constraints when no more wave 1 predictors would improve the fit of the model.

#### Hypothesized direct effects on wave 6 latent reckless or inattentive driving

As hypothesized, in model 3, wave 1 movie reckless driving had a significant positive effect on reckless driving *(β* = .074, *p* = .014) and a non-significant negative effect on inattentive driving (*β* = −.035, *p* = .354) and the difference in effects was significant. (χ^2^ (1) = 7.57, *N* = 1,630, *p* = .006). As hypothesized, wave 1 self-regulation had a significant negative impact on inattentive driving (*β* = −.145, *p* = .002) but a non-significant negative impact on reckless driving (*β* = −.052, *p* = .163) and the difference in effects was marginally significant (χ^2^ (1) = 3.64, *N* = 1630, *p* = .056).

For the post hoc tests, age predicted reckless driving (*β* = .212, *p*<.001) but not inattentive driving (*β* = .070, *p* = .070) and the effects were significantly different (χ^2^ (1) = 12.62, *N* = 1630, *p*<.001). All other predictors similarly affected reckless and inattentive driving, of which socioeconomic status (SES) and sensation seeking had the strongest effects (*β* = .224, *p*<.001; *β* = .218, *p*<.001, respectively), followed by extracurricular activities, which was negatively related to both constructs (*β* = −.075, *p* = .010). Contrary to our hypothesis, there was no evidence that the effect of sensation seeking was different for reckless driving as compared to inattentive driving. Thus, we could not reject the hypothesis of equal magnitude effects on both reckless and inattentive driving (χ^2^ (1) = 0.13, N = 1630, *p* = .722). The same pattern of results held true for the effects of wave 4 sensation seeking. These steps led to a better fit for model 3; χ^2^ [df = 142, n = 1630] = 247.80, CFI  = .944, RMSEA  = .021. The nested chi-square for model 3 compared to the model 2 was 24.04, df = 3, *p*<.001.

In the final step, in model 4 we investigated specific direct effects of w1 predictors, over and above their indirect influence through the latent constructs on individual risky driving indicators. We used a more stringent p value of.001 for significance for these post hoc tests. Lack of seatbelt use was predicted by sensation seeking (*β* = .18, *p*<.01) and by male gender (*β* = .13, *p*<.01). Adding these two effects to the previous model, model 3, did not alter the pattern of significance for the other parameters. The chi-square for the model was 212.67, *df* = 140, *n* = 1630, CFI  = .962, RMSEA  = .018. The nested chi-square for comparison of model 4 to the model 3 was 37.04, *df* = 2, *p*<.001.

In summary, we added 6 parameters to our initial simplest model, 3 to test a priori hypotheses and 3 based on post hoc inspection of results to arrive at the final model. The overall improvement in model fit was highly significant (nested chi-square  = 99.92, *df* = 6, *p*<.001).

Complete results of the final direct effects model are shown in S4 and S5 Tables.

#### Indirect effects model

In this model, we investigated the potential mediating role of the personality trait sensation seeking (at wave 4). To test our prediction that reckless driving was not only directly, but also indirectly affected by exposure to movie reckless driving through wave 4 sensation seeking, we added pathways in the model between wave 1 predictors and wave 4 sensation seeking, and between wave 4 sensation seeking and reckless driving and inattentive driving. This model fit the data well, χ^2^ [*df* = 148, *n* = 1,630] = 238.903, CFI  = .960, RMSEA  = .019]. The effect of w4 sensation seeking on reckless driving and inattentive driving was strongly significant (*β* = .09, *p*<.001) and we found no evidence that the effect on reckless driving was different than the effect on inattentive driving.

#### Hypothesized direct effects on w4 sensation seeking

Not surprisingly, wave 1 sensation seeking was strongly related to wave 4 sensation seeking (*β* = .46, *p*<.001). Contrary to our hypothesis, the wave 1 movie reckless driving effect on wave 4 sensation seeking was not significant (*β* = .03, NS.). Consistent with previous research, ^35^ but not a focus of this work, we found that the number of movies per week was a significant predictor of wave 4 sensation seeking (*β* = .06, *p* = .005). Other predictors with significant effects on wave 4 sensation seeking included: male gender (*β* = .05, *p* = .04), lower SES (*β* = −.08, *p* = .007), higher rebelliousness (*β* = .06, *p* = .01), and lower parental support (*β* = −.06, *p* = .005).

#### Hypothesized indirect effects on w6 latent reckless driving through w4 sensation seeking

Contrary to our hypothesis, the indirect effect of wave 1 movie reckless driving on wave 6 reckless driving through wave 4 sensation seeking was not significant (*β* = .004, *p* = .171). As noted previously, however, the direct effect was significant.

Although it is not the focus of this work, because we have shown in previously published work that the number of wave 1 R-rated movies viewed significantly predicted growth in sensation seeking from wave 1 to wave 4 [35], we decided to examine the indirect effect of movies per week on reckless and inattentive driving through w4 sensation seeking. The indirect effect was significant (*β* = .007, *p* = .023).

#### Non-hypothesized direct effects on w6 indicators of reckless or inattentive driving

Male gender and wave 1 sensation seeking had significant positive effects on failure to use seatbelts. With the addition of wave 4 sensation seeking to the model, we decided to perform an additional post hoc test of its effect on failure to use seatbelts and found that the effect was positive and significant (*β* = .136, *p*<.001). The effect for wave 1 sensation seeking was reduced slightly but still strongly significant (*β* = .119, *p* = .003) and the effect of male gender increased slightly (*β* = .125, *p*<.001). No other effects were significant at *p*<.001.

To put the effect sizes for the hypothesized associations on wave 6 reckless driving into perspective, we re-ran the final model using logistic regressions (for the connections between the wave 6 indicators and the wave 6 latent variables) to obtain odds ratios (OR) for the indirect effects of wave 1 predictors on the individual wave 6 reckless driving items. Results are shown in Table 2. The OR contrasts a high to a low score on the wave 1 risk factor (95^th^ compared to 5^th^ percentile). The indirect effects are either through just the wave 6 reckless driving latent factor or through both wave 4 sensation seeking and then the wave 6 reckless driving factor (double indirect). Consistent with the lack of direct effect of wave 1 movie reckless driving on wave 4 sensation seeking, only the direct effects of wave 1 movie reckless driving on the wave 6 reckless driving items were significant and the adjusted odds ratios ranged from 1.16 to 1.98. In contrast, wave 1 sensation seeking had both significant indirect effects ranging from 1.55 to 3.37 and significant double indirect effects ranging from 1.12 to 1.59. Effects of sensation seeking were uniformly larger than the corresponding effects for movie reckless driving.

**Table 2:** Odds Ratios for the indirect and double indirect effects of wave 1 high movie reckless driving exposure and sensation seeking (95th percentile) as compared to low movie reckless driving exposure and sensation seeking (5th percentile) on the separate wave 6 reckless driving items.

	Movie Reckless Driving (w1)						Sensation Seeking (w1)					
	Indirect			Double Indirect			Indirect			Double Indirect		
	OR	95% CI		OR	95% CI		OR	95% CI		OR	95% CI	
Over Speed Limit	1.976	1.085	3.599	1.027	0.986	1.071	3.374	1.647	6.913	1.381	1.060	1.800
Tailgated	1.602	1.072	2.394	1.019	0.992	1.048	2.319	1.477	3.639	1.251	1.045	1.498
Weaved in and out of traffic	1.484	1.060	2.079	1.016	0.993	1.040	2.024	1.392	2.943	1.207	1.034	1.409
Cross double yellow line to pass	1.278	1.034	1.579	1.010	0.994	1.026	1.550	1.222	1.964	1.124	1.017	1.242
Sped through a yellow light	1.540	1.064	2.231	1.017	0.992	1.043	2.162	1.435	3.256	1.228	1.035	1.456
Failed to use seatbelt	1.160	1.013	1.327	1.006	0.996	1.016	2.784	1.841	4.210	1.589	1.306	1.933

w1  =  wave 1, OR  =  odds ratios, CI  =  confidence interval.

## Discussion

This study demonstrates a direct long-term relation between early exposure to reckless driving in movies and reckless driving behaviors among US adolescents with driving experience. Moreover, this relation was evident while controlling for many other important background variables. In contrast, movie reckless driving exposure was not associated with inattentive driving. Although the long-term relation between reckless driving and video game play has been established [19], [20] this study is the first, to our knowledge, to show a direct long-term relation between passive exposure to reckless driving content in movies and reckless driving among adolescents. Although not specifically tested, these findings are in line with early socio-cognitive models of learning through experience or observation [22], [55] the notion that repeated exposure to risk-glorifying media may instigate risk taking behaviors by the activation of positive risk-related cognitions, beliefs and behavioral scripts [23] and additionally, through changes in the self-concept related to risk-tasking [21].

The main finding, suggesting that exposure to movie reckless driving may program behaviors scripts for later reckless driving raises questions about what might be done. Firstly, movie producers should be aware that behaviors depicted in movies can influence adolescent behaviors. This has been shown decisively for movie smoking, for which the US Surgeon General states that exposure is one cause of adolescent smoking [56]. Movie smoking is now incorporated into some ratings systems but reckless driving is not—it could be. Parents may also play a role, as many previous research studies have found that parental R-rated movie restriction is associated with lower rates of adolescent substance use [57]–[61], presumably as a result of decreased exposure. Parents should understand that adolescents model behaviors they see in movies and should restrict children and adolescents in the number and types of movies they are allowed to watch each week.

Previous research was replicated by showing that high sensation seeking tendencies are important risk-factors for unsafe driving behaviors [8], [29]. Moreover, weekly frequency of movie exposure was indirectly related to these behaviors through sensation seeking. These results are consistent with the conclusion that reckless driving in movies directly impacted adolescent future reckless driving practices, whereas frequent overall screen exposure may have stimulated reckless driving through exposure to a variety of other risk-taking behaviors such as excessive drinking, movie violence and their cumulative impact on sensation seeking tendencies [35], [62] Previous research indicates that adolescents who frequently watch R-rated movies, rated such for portraying higher levels of risk taking behavior and violence [35] show increases in sensation seeking over time [63]. Adolescents with poor self-regulation reported higher rates of inattentive but not (intentional) reckless driving. This finding is in line with the notion that the capacity to maintain focused attention is an important mechanism underlying self-regulatory functioning [33]. A major distraction mentioned by teenage drivers is the presence of passengers [64], which has been shown to increase the risk of fatal crashes among 16 and 17 year-old drivers [65]. Inexperienced drivers may be easily distracted; graduated licensing laws may prohibit newly licensed drivers from carrying passengers.

Conforming with prior literature [9], [66], males and adolescents with high scores on sensation seeking were less inclined to use seatbelts than their female and lower sensation seeking counterparts. Although not using a seatbelt could be inattentive, these results suggest that it is volitional. Since seatbelt use appears to be one of the most effective measures to reduce injuries in motor vehicle crashes [67], interventions to use seatbelts should be aimed at males and high risk takers in particular, and based on the premise that nonuse is volitional rather than forgetful.

One of the potential limitations of this longitudinal study is attrition bias due to eligible teens (with driving experience) who dropped out before wave 5. Although we know how many teens dropped out by wave 5, we do not know which of the dropouts had driving experience. If the relations among the study variables are quite different for these teens, our results could be biased compared to the full population.

Another issue that should be mentioned here is the omission of a potentially important covariate, namely, number of miles driven during the year [68]. We selected only teens with driving experience by wave 5 for inclusion to make sure that by wave 6, all teens included in the analysis could report on a full year of driving exposure, but this does not necessarily mean that all teens drove the same number of miles in the exposure year. The probability of endorsing some of the unsafe driving items could be increased by miles driven, simply because more driving allows for more opportunities to engage in unsafe (reckless or inattentive) driving behaviors. Indeed, population crash rates are different when miles driven are figured into the denominator [69]. To some extent, this might explain why older age and higher SES (both of which are proxies for miles driven [68], [70], [71]) had relatively strong positive effects on unsafe driving. The fact that we controlled for proxies for miles driven (age and SES) and selected only those with driving experience by wave 5 should mitigate these concerns to some extent.

The strength of this study is that a unique part of adolescents’ reckless driving was demonstrated to be affected by exposure to reckless driving in movies, exposure that was measured well before these adolescents ever got behind the wheel of a car. This association was found to be independent of various potential confounding variables (e.g., the reported frequency of watching movies) and sensation seeking tendencies. Therefore, the results of this study are consistent with the idea that seeing reckless driving on screen is one cause of reckless driving. More observational and experimental studies are needed to make a definitive causal statement in this area.

Finally, this study demonstrates the importance of making a distinction between adolescents’ reckless driving and inattentive driving because each requires different prevention strategies. Inattentive driving is related to poor self-regulation, suggesting that attention deficits and high distractibility are substantial risk factors of unsafe driving practices among adolescents. Reckless driving is related to risk taking propensity, male gender, videogame use and exposure to reckless driving in movies. Interventional researchers should address both issues and related risks.

## Conclusions

Besides replicating previous findings on well-known unsafe driving-related risk-factors such as male gender and sensation seeking [8], [9], this study shows that exposure to reckless driving in movies may increase future tendencies to drive recklessly in adolescents. Whereas risky health behaviors such as excessive smoking or drinking primarily pose a threat to one’s own health, risk taking in traffic may also potentially harm others. In light of traffic injury prevention, the specific mechanisms that make adolescents drive more recklessly when they have been exposed to reckless driving in movies should be further examined.

## Supporting Information

S1 Table **Overview of wave 1 control variables.**
(DOCX)

S2 Table **Descriptive statistics for adolescent and social-environmental characteristics.**
(DOCX)

S3 Table **Correlations of wave 1 predictors with wave 6 unsafe driving composites.**
(DOCX)

S4 Table **Parameter estimates for measurement portion of final direct effects model.**
(DOCX)

S5 Table **Parameter estimates for structural portion of final direct effects model.**
(DOCX)

S6 Table **Estimates for probit indirect effects model.**
(DOCX)

S7 Table **Estimates for logit indirect effects model.**
(DOCX)
