# Supplementary material for: Exposure to Movie Reckless Driving in Early Adolescence Predicts Reckless, but Not Inattentive Driving
Source: PLoS One. 2014 Dec 10;9(12):e113927. doi: 10.1371/journal.pone.0113927 (PMC4262265; doi:10.1371/journal.pone.0113927)
Supplement: S1 Table — Overview of wave 1 control variables. (DOCX) [file pone.0113927.s001.docx]

**Table S1. Overview of Wave 1 Control Variables.**

| Variable | Survey Question(s) | Response Options |
| --- | --- | --- |
| Parent education ^a^ | What is the highest grade or year of school that you completed? | Up to 8^th^ grade 9^th^ to 11^th^ grade 12^th^ grade but no diploma  HS diploma/ equivalent Voc/Tech after HS, no diploma Voc/Tech program after HS Some college but no degree Associates degree (A.A., A.S.) Bachelor’s degree (B.A., B.S.) Some grad/professional, no degree Master’s degree (M.A., M.S.) Professional degree beyond bachelors (MD, DDS, JD, LLB) |
| Household income ^a^ | In studies like this, households are sometimes grouped according to income. Please tell me which group best describes the total income of all persons living in this household over the past year? Please include income from all sources, such as salaries, interest, retirement, or any other source for all household members. Would you say… | ≤ $10 000 $10 000-$20 000 $20 000- $30 000 $30 000- $50 000 $50 000-$75 000 ≥ $75 000 |
| School performance | How would you describe your grades in school? | Below average Average Good Excellent |
| Watched movies per week | About how many movies do you usually watch each week? Please include movies you see in movie theatres, on videotape or DVD, and on television. | None One to two Three to four Five or more |
| Television exposure per day | On school days, how many hours a day do you usually watch TV? Please do not include the time  you use the TV to play video games. | None Less than one hour  Three to four hours More than four hours |
| Hours a day spent playing video or computer games | On school days, how many hours a day do you usually spend playing video or computer games? | None  Less than one hour  Three to four hours  More than four hours |
| Parental support (9-item index; Cronbach’s *a* = .74). | She/He is pleased with how I behave  She/He listens to what I have to say  She/He makes me feel better when I am upset  She/He wants to hear about my problems  She/He likes me just the way I am  She/He is too busy to talk to me  She/He makes rules without asking what I think (reversed)  She/He is always telling me what to do (reversed)  She/He tells me when I do a good job on things | Not like him/her  A little like him/her  A lot like him/her  Just like him/her |
| Parental Control (7-item index; Cronbach’s *a* = .72) | She/He checks to see if I do my homework She/He makes sure I tell her/him where I’m going  She/He knows where I am after school She/He tells me times when I must come home She/He has rules that I must follow She/He makes sure I go to bed on time She/He asks me what I do with my friends | Not like him/her  A little like him/her  A lot like him/her  Just like him/her |
| Extracurricular activities | How often do you participate in team sports were there is a coach? How often do you participate in other sports without a coach? How often do you attend church or other religious activities? How often do you go to music lessons, choir, dance, or band practice? How often do you participate in school clubs or activities like math or science clubs or the school paper? How often do you participate in other clubs like the Boy or Girl Scouts, 4-H, or the Boys or Girls Clubs of America? | Almost everyday  1 to a few times a week  1 to a few times a month  Never |
| Sensation seeking (4-item index; Cronbach’s *a* = .59) | I like to do scary things I like to do dangerous things I often think there is nothing to do I like to listen to loud music | Not like you A little like you A lot like you Just like you |
| Rebelliousness (6-item index; Cronbach’s α = .69) | I get in trouble in school I argue a lot with other kids I do things my parents wouldn’t want me to do I do what my teachers tell me to do (reversed) I argue with my teachers I like to break the rules | Not like you A little like you A lot like you Just like you |
| Self regulation (4-item index; Cronbach’s α = .41) | I am good at waiting my turn I get my homework done first so I can have fun later I bother other students when they are trying to work I have to be reminded several times to do things | Not like you A little like you A lot like you Just like you |

^a^ Refers to items that were combined to compute socioeconomic status
