# Supplementary material for: Exposure to Movie Reckless Driving in Early Adolescence Predicts Reckless, but Not Inattentive Driving
Source: PLoS One. 2014 Dec 10;9(12):e113927. doi: 10.1371/journal.pone.0113927 (PMC4262265; doi:10.1371/journal.pone.0113927)
Supplement: S2 Table — Descriptive statistics for adolescent and social-environmental characteristics. (DOCX) [file pone.0113927.s002.docx]

**Table S2. Descriptive Statistics for Adolescent and Social-Environmental Characteristics**

| Characteristic | Total (*n* = 1647) |
| --- | --- |
| Prevalence (W1) |  |
| **Age (years)**  10 11 12 13 14 | 18% 20% 21% 21% 20% |
| **Parent education**  Very low (1-3)  Low (4)  Average (5-7)  High (8-9)  Very high (10-13) | 17% 23% 21% 27% 12% |
| **Household income (per year)**  ≤ $10 000  $10 000-$20 000  $20 000- $30 000  $30 000- $50 000  $50 000-$75 000  ≥ $75 000 | 8% 10% 12% 21% 19% 30% |
| **School performance**  Average or below  Good  Excellent | 22% 41% 37% |
| **Watched movies per week**  None  One to two  Three to four  Five or more | 3%  39%  31%  28% |
| **Television exposure per day**  None  Less than one hour  One to two hours  Three to four hours  More than four hours | 6%  21%  48%  19%  6% |
| **Hours a day spent playing video/computer games**  None  Less than one hour  One to two hours  Three to four hours  More than four hours | 29%  39%  25% 5% 2% |
| Mean (Standard Deviation)  **Parenting style**  Responsiveness  Demandingness | 3.30 (.45)  3.33 (.48) |
| **SES ^a^** | .32 (.65) |
| **Extracurricular Activities** | 2.82 (.48) |
| **Sensation seeking** | 1.96 (.59) |
| **Rebelliousness** | 1.33 (.43) |
| **Self-regulation** | 2.99 (.47) |

The scores for sensation seeking, rebelliousness, self-regulation and the parenting style subscales ranged between 1 and 4, with higher scores reflecting higher average levels on these scales.

^a^ SES was determined by combining the variables parent education and household income into a standardized score, ranging between -.1.85 and 1.39, with higher values indicating higher socioeconomic status.
