# Supplementary material for: Exposure to Movie Reckless Driving in Early Adolescence Predicts Reckless, but Not Inattentive Driving
Source: PLoS One. 2014 Dec 10;9(12):e113927. doi: 10.1371/journal.pone.0113927 (PMC4262265; doi:10.1371/journal.pone.0113927)
Supplement: S3 Table — Correlations of wave 1 predictors with wave 6 unsafe driving composites. (DOCX) [file pone.0113927.s003.docx]

| **Table S3. Correlations of wave 1 predictors with wave 6 unsafe driving composites.** | | | |
| --- | --- | --- | --- |
|  | **Unsafe** | **Reckless** | **Inattentive** |
| Movie Reckless Driving | 0.05* | 0.07** | -0.01 |
| Self-regulation | -0.13*** | -0.10*** | -0.12*** |
| Age | 0.18*** | 0.20*** | 0.08** |
| Sensation Seeking | 0.22*** | 0.22*** | 0.14*** |
| Socioeconomic Status | 0.13*** | 0.12*** | 0.08** |
| Extracurricular Activities | -0.05 | -0.05* | -0.02 |
| Male Gender | 0.08** | 0.07** | 0.05* |
| Parent Education | 0.06* | 0.06* | 0.04 |
| School Performance | -0.05 | -0.04 | -0.04 |
| Rebelliousness | 0.11*** | 0.09*** | 0.09*** |
| Movies Per Week | 0.05 | 0.06* | 0.01 |
| TV Hours Per Day | 0.00 | 0.01 | -0.02 |
| Video Games Per Day | 0.01 | 0.02 | 0.00 |
| Parental Support | -0.08** | -0.08*** | -0.03 |
| Parental Control | -0.09*** | -0.10*** | -0.03 |
| Unsafe driving is based on all 9 items. Pairwise sample size varies between 1642 and 1647 due to missing data. * .01 < *p* <= .05, ** .001 < *p* <= .01, *** 0 < *p* <= .001 | | | |
