# Supplementary material for: Exposure to Movie Reckless Driving in Early Adolescence Predicts Reckless, but Not Inattentive Driving
Source: PLoS One. 2014 Dec 10;9(12):e113927. doi: 10.1371/journal.pone.0113927 (PMC4262265; doi:10.1371/journal.pone.0113927)
Supplement: S4 Table — Parameter estimates for measurement portion of final direct effects model. (DOCX) [file pone.0113927.s004.docx]

| **Table S4. Parameter estimates for measurement portion of final direct effects model.** | | | |
| --- | --- | --- | --- |
|  | Estimate | Est./S.E. | *p* |
| **Factor Loadings Reckless Driving** |  |  |  |
| Speeding | 0.784 | 19.347 | 0.000 |
| Tailgating | 0.680 | 22.970 | 0.000 |
| Weaving In and Out | 0.624 | 20.324 | 0.000 |
| Cross Double Yellow Line | 0.472 | 11.573 | 0.000 |
| Speed through Yellow Light | 0.679 | 20.836 | 0.000 |
| Fail to Use Seatbelts | 0.372 | 9.826 | 0.000 |
| **Factor Loadings Inattentive Driving** |  |  |  |
| Fail to Yield | 0.755 | 17.764 | 0.000 |
| Run Red Light | 0.493 | 11.813 | 0.000 |
| Ignore Stop Sign | 0.599 | 14.377 | 0.000 |
| **Indicator Thresholds** |  |  |  |
| Speeding | 1.624 | 2.044 | 0.041 |
| Tailgating | 1.732 | 2.427 | 0.015 |
| Weaving In and Out | 1.618 | 2.196 | 0.028 |
| Cross Double Yellow Line | 1.344 | 1.663 | 0.096 |
| Speed through Yellow Light | 1.186 | 1.695 | 0.090 |
| Fail to Use Seatbelts | 0.568 | 0.789 | 0.430 |
| Fail to Yield | 0.838 | 1.120 | 0.263 |
| Run Red Light | 1.280 | 1.744 | 0.081 |
| Ignore Stop Sign | 1.339 | 1.727 | 0.084 |
| **Correlations** |  |  |  |
| Reckless with Inattentive Driving | 0.669 | 15.943 | 0.000 |
| Speed through Yellow Light with Run Red Light | 0.259 | 5.430 | 0.000 |
