# Supplementary material for: Exposure to Movie Reckless Driving in Early Adolescence Predicts Reckless, but Not Inattentive Driving
Source: PLoS One. 2014 Dec 10;9(12):e113927. doi: 10.1371/journal.pone.0113927 (PMC4262265; doi:10.1371/journal.pone.0113927)
Supplement: S5 Table — Parameter estimates for structural portion of final direct effects model. (DOCX) [file pone.0113927.s005.docx]

| **Table S5. Parameter estimates for structural portion of final direct effects model.** | | | | | | | | | |
| --- | --- | --- | --- | --- | --- | --- | --- | --- | --- |
|  | **Reckless Driving** | | | **Inattentive Driving** | | | **Fail to Use Seatbelts** | | |
| **W1 Predictor Effects** | Estimate | Est./S.E. | *p* | Estimate | Est./S.E. | *p* | Estimate | Est./S.E. | *p* |
| Movie Reckless Driving | 2.016 | 2.438 | 0.015 | -0.923 | -0.926 | 0.354 |  |  |  |
| Self-Regulation | -0.121 | -1.389 | 0.165 | -0.331 | -3.126 | 0.002 |  |  |  |
| Age | 0.180 | 6.768 | 0.000 | 0.058 | 1.811 | 0.070 |  |  |  |
| Sensation Seeking | 0.360 | 5.979 | 0.000 | 0.360 | 5.979 | 0.000 | 0.320 | 4.837 | 0.000 |
| SES | 0.377 | 5.248 | 0.000 | 0.377 | 5.248 | 0.000 |  |  |  |
| Extracurricular Activities | -0.166 | -2.579 | 0.010 | -0.166 | -2.579 | 0.010 |  |  |  |
| Male Gender | 0.104 | 1.649 | 0.099 | 0.104 | 1.649 | 0.099 | 0.278 | 3.711 | 0.000 |
| Parent Education | -0.067 | -1.731 | 0.084 | -0.067 | -1.731 | 0.084 |  |  |  |
| School Performance | 0.025 | 0.588 | 0.556 | 0.025 | 0.588 | 0.556 |  |  |  |
| Rebelliousness | -0.037 | -0.467 | 0.640 | -0.037 | -0.467 | 0.640 |  |  |  |
| Movies per Week | 0.037 | 1.030 | 0.303 | 0.037 | 1.030 | 0.303 |  |  |  |
| TV Hours per Day | -0.026 | -0.785 | 0.433 | -0.026 | -0.785 | 0.433 |  |  |  |
| Video Games Hours per Day | -0.058 | -1.695 | 0.090 | -0.058 | -1.695 | 0.090 |  |  |  |
| Parental Support | 0.054 | 0.712 | 0.477 | 0.054 | 0.712 | 0.477 |  |  |  |
| Parent Control | -0.018 | -0.256 | 0.798 | -0.018 | -0.256 | 0.798 |  |  |  |
