# Supplementary material for: Exposure to Movie Reckless Driving in Early Adolescence Predicts Reckless, but Not Inattentive Driving
Source: PLoS One. 2014 Dec 10;9(12):e113927. doi: 10.1371/journal.pone.0113927 (PMC4262265; doi:10.1371/journal.pone.0113927)
Supplement: S6 Table — Estimates for probit indirect effects model. (DOCX) [file pone.0113927.s006.docx]

| **Table S6. Estimates for probit indirect effects model.** | | | | | |
| --- | --- | --- | --- | --- | --- |
|  | Estimate | Est./S.E. | *p* |  | Std. Estimate |
| **Reckless Driving** |  |  |  |  |  |
| Over Speed Limit | 0.779 | 19.306 | 0.000 |  | 0.806 |
| Tailgated | 0.677 | 22.823 | 0.000 |  | 0.709 |
| Weaved in and out of traffic | 0.623 | 20.304 | 0.000 |  | 0.656 |
| Cross double yellow line to pass | 0.474 | 11.658 | 0.000 |  | 0.506 |
| Sped through a yellow light | 0.676 | 20.838 | 0.000 |  | 0.708 |
| Failed to use seatbelt | 0.359 | 9.437 | 0.000 |  | 0.368 |
| **Inattentive Driving** |  |  |  |  |  |
| Failed to yield | 0.760 | 17.817 | 0.000 |  | 0.783 |
| Ran red light | 0.479 | 11.493 | 0.000 |  | 0.504 |
| Ignored stop sign | 0.597 | 14.482 | 0.000 |  | 0.623 |
| **Reckless Driving on** |  |  |  |  |  |
| Age | 0.183 | 6.872 | 0.000 |  | 0.217 |
| Male Gender | 0.095 | 1.493 | 0.135 |  | 0.043 |
| Parent Education | -0.066 | -1.711 | 0.087 |  | -0.073 |
| School Performance | 0.029 | 0.671 | 0.502 |  | 0.020 |
| SES | 0.397 | 5.477 | 0.000 |  | 0.236 |
| Rebelliousness | -0.054 | -0.679 | 0.497 |  | -0.021 |
| Self-regulation | -0.111 | -1.278 | 0.201 |  | -0.047 |
| Movies per week | 0.027 | 0.769 | 0.442 |  | 0.022 |
| TV hours per day | -0.029 | -0.882 | 0.378 |  | -0.025 |
| Video games hours per day | -0.057 | -1.660 | 0.097 |  | -0.050 |
| Extracurricular Activities | -0.163 | -2.517 | 0.012 |  | -0.072 |
| Parental Support | 0.074 | 0.971 | 0.332 |  | 0.030 |
| Parental Control | -0.011 | -0.158 | 0.875 |  | -0.005 |
| Sensation Seeking wave 1 | 0.293 | 3.942 | 0.000 |  | 0.159 |
| Movie Reckless driving exposure | 1.922 | 2.320 | 0.020 |  | 0.071 |
| Sensation seeking wave 4 | 0.169 | 2.676 | 0.007 |  | 0.097 |
| **Inattentive Driving on** |  |  |  |  |  |
| Age | 0.061 | 1.899 | 0.058 |  | 0.073 |
| Male Gender | 0.095 | 1.493 | 0.135 |  | 0.044 |
| Parent Education | -0.066 | -1.711 | 0.087 |  | -0.075 |
| School Performance | 0.029 | 0.671 | 0.502 |  | 0.020 |
| SES | 0.397 | 5.477 | 0.000 |  | 0.242 |
| Rebelliousness | -0.054 | -0.679 | 0.497 |  | -0.021 |
| Self-regulation | -0.321 | -3.026 | 0.002 |  | -0.140 |
| Movies per week | 0.027 | 0.769 | 0.442 |  | 0.022 |
| TV hours per day | -0.029 | -0.882 | 0.378 |  | -0.026 |
| Video games hours per day | -0.057 | -1.660 | 0.097 |  | -0.051 |
| Extracurricular Activities | -0.163 | -2.517 | 0.012 |  | -0.073 |
| Parental Support | 0.074 | 0.971 | 0.332 |  | 0.031 |
| Parental Control | -0.011 | -0.158 | 0.875 |  | -0.005 |
| Sensation Seeking wave 1 | 0.227 | 2.579 | 0.010 |  | 0.126 |
| Movie Reckless driving exposure | -1.047 | -1.050 | 0.294 |  | -0.039 |
| Sensation seeking wave 4 | 0.227 | 3.128 | 0.002 |  | 0.133 |
| **Failed to use seatbelt** |  |  |  |  |  |
| Sensation Seeking wave 1 | 0.202 | 2.755 | 0.006 |  | 0.113 |
| Male Gender | 0.266 | 3.570 | 0.000 |  | 0.125 |
| Sensation seeking wave 4 | 0.243 | 3.878 | 0.000 |  | 0.143 |
| **Sensation seeking wave 4** |  |  |  |  |  |
| Age | -0.011 | -1.092 | 0.275 |  | -0.023 |
| Male Gender | 0.056 | 2.028 | 0.043 |  | 0.045 |
| Parent Education | -0.002 | -0.124 | 0.902 |  | -0.004 |
| School Performance | -0.015 | -0.785 | 0.432 |  | -0.018 |
| SES | -0.080 | -2.709 | 0.007 |  | -0.083 |
| Rebelliousness | 0.081 | 2.528 | 0.011 |  | 0.055 |
| Self-regulation | -0.047 | -1.499 | 0.134 |  | -0.035 |
| Movies per week | 0.044 | 2.785 | 0.005 |  | 0.061 |
| TV hours per day | 0.014 | 1.000 | 0.317 |  | 0.021 |
| Video games hours per day | -0.007 | -0.463 | 0.644 |  | -0.010 |
| Extracurricular Activities | -0.018 | -0.646 | 0.518 |  | -0.014 |
| Parental Support | -0.088 | -2.769 | 0.006 |  | -0.064 |
| Parental Control | -0.031 | -1.019 | 0.308 |  | -0.023 |
| Sensation Seeking wave 1 | 0.483 | 19.856 | 0.000 |  | 0.460 |
| Movie Reckless driving exposure | 0.480 | 1.503 | 0.133 |  | 0.031 |
| **Correlation:** Reckless driving with inattentive driving | 0.660 | 15.555 | 0.000 |  | 0.660 |
| **Correlation:** Sped through yellow with run red light | 0.266 | 5.574 | 0.000 |  | 0.266 |
| **Intercepts** |  |  |  |  |  |
| Sensation seeking wave 4 | 1.593 | 6.248 | 0.000 |  | 2.558 |
| **Thresholds** |  |  |  |  |  |
| Over Speed Limit | 1.833 | 2.306 | 0.021 |  | 1.741 |
| Tailgated | 1.914 | 2.664 | 0.008 |  | 1.839 |
| Weaved in and out of traffic | 1.785 | 2.414 | 0.016 |  | 1.725 |
| Cross double yellow line to pass | 1.471 | 1.813 | 0.070 |  | 1.442 |
| Sped through a yellow light | 1.368 | 1.947 | 0.051 |  | 1.315 |
| Failed to use seatbelt | 1.053 | 1.462 | 0.144 |  | 0.991 |
| Failed to yield | 1.114 | 1.487 | 0.137 |  | 1.076 |
| Ran red light | 1.453 | 1.969 | 0.049 |  | 1.432 |
| Ignored stop sign | 1.555 | 2.003 | 0.045 |  | 1.522 |
| **Residual Variances** |  |  |  |  |  |
| Sensation seeking wave 4 | 0.256 | 28.268 | 0.000 |  | 0.659 |
| Reckless driving | 1.000 |  |  |  | 0.842 |
| Inattentive driving | 1.000 |  |  |  | 0.880 |
| **R Squared** |  |  |  |  |  |
| Over Speed Limit |  |  |  |  | 0.649 |
| Tailgated |  |  |  |  | 0.503 |
| Weaved in and out of traffic |  |  |  |  | 0.431 |
| Cross double yellow line to pass |  |  |  |  | 0.256 |
| Sped through a yellow light |  |  |  |  | 0.502 |
| Failed to use seatbelt |  |  |  |  | 0.249 |
| Failed to yield |  |  |  |  | 0.613 |
| Ran red light |  |  |  |  | 0.254 |
| Ignored stop sign |  |  |  |  | 0.388 |
