# Supplementary material for: Exposure to Movie Reckless Driving in Early Adolescence Predicts Reckless, but Not Inattentive Driving
Source: PLoS One. 2014 Dec 10;9(12):e113927. doi: 10.1371/journal.pone.0113927 (PMC4262265; doi:10.1371/journal.pone.0113927)
Supplement: S7 Table — Estimates for logit indirect effects model. (DOCX) [file pone.0113927.s007.docx]

| **Table S7. Estimates for logit indirect effects model.** | | | |
| --- | --- | --- | --- |
|  |  |  |  |
|  | Estimate | Est./S.E. | *p* |
|  |  |  |  |
| **Reckless Driving** |  |  |  |
| Over Speed Limit | 2.397 | 7.354 | 0.000 |
| Tailgated | 1.657 | 10.986 | 0.000 |
| Weaved in and out of traffic | 1.391 | 11.127 | 0.000 |
| Cross double yellow line to pass | 0.864 | 8.105 | 0.000 |
| Sped through a yellow light | 1.521 | 10.528 | 0.000 |
| Failed to use seatbelt | 0.523 | 6.563 | 0.000 |
| **Inattentive Driving** |  |  |  |
| Failed to yield | 1.871 | 7.834 | 0.000 |
| Ran red light | 0.867 | 7.996 | 0.000 |
| Ignored stop sign | 1.300 | 8.403 | 0.000 |
| **Reckless Driving on** |  |  |  |
| Age | 0.189 | 7.206 | 0.000 |
| Male Gender | 0.093 | 1.479 | 0.139 |
| Parent Education | -0.073 | -1.828 | 0.068 |
| School Performance | 0.036 | 0.808 | 0.419 |
| SES | 0.442 | 5.905 | 0.000 |
| Rebelliousness | -0.046 | -0.571 | 0.568 |
| Self-regulation | -0.116 | -1.355 | 0.175 |
| Movies per week | 0.029 | 0.827 | 0.408 |
| TV hours per day | -0.029 | -0.856 | 0.392 |
| Video games hours per day | -0.059 | -1.759 | 0.079 |
| Extracurricular Activities | -0.165 | -2.584 | 0.010 |
| Parental Support | 0.090 | 1.235 | 0.217 |
| Parental Control | -0.017 | -0.237 | 0.813 |
| Sensation Seeking wave 1 | 0.290 | 3.818 | 0.000 |
| Movie Reckless driving exposure | 1.880 | 2.324 | 0.020 |
| Sensation seeking wave 4 | 0.160 | 2.448 | 0.014 |
| **Inattentive Driving on** |  |  |  |
| Age | 0.059 | 1.844 | 0.065 |
| Male Gender | 0.093 | 1.479 | 0.139 |
| Parent Education | -0.073 | -1.828 | 0.068 |
| School Performance | 0.036 | 0.808 | 0.419 |
| SES | 0.442 | 5.905 | 0.000 |
| Rebelliousness | -0.046 | -0.571 | 0.568 |
| Self-regulation | -0.362 | -3.685 | 0.000 |
| Movies per week | 0.029 | 0.827 | 0.408 |
| TV hours per day | -0.029 | -0.856 | 0.392 |
| Video games hours per day | -0.059 | -1.759 | 0.079 |
| Extracurricular Activities | -0.165 | -2.584 | 0.010 |
| Parental Support | 0.090 | 1.235 | 0.217 |
| Parental Control | -0.017 | -0.237 | 0.813 |
| Sensation Seeking wave 1 | 0.247 | 2.844 | 0.004 |
| Movie Reckless driving exposure | -1.208 | -1.205 | 0.228 |
| Sensation seeking wave 4 | 0.236 | 3.095 | 0.002 |
| **Failed to use seatbelt on** |  |  |  |
| Sensation Seeking wave 1 | 0.459 | 3.820 | 0.000 |
| Male Gender | 0.433 | 3.699 | 0.000 |
| Sensation seeking wave 4 | 0.465 | 4.083 | 0.000 |
| **Sensation seeking wave 4** |  |  |  |
| Age | -0.011 | -1.067 | 0.286 |
| Male Gender | 0.056 | 2.000 | 0.045 |
| Parent Education | -0.001 | -0.077 | 0.938 |
| School Performance | -0.011 | -0.560 | 0.575 |
| SES | -0.073 | -2.234 | 0.025 |
| Rebelliousness | 0.086 | 2.015 | 0.044 |
| Self-regulation | -0.046 | -1.328 | 0.184 |
| Movies per week | 0.044 | 2.804 | 0.005 |
| TV hours per day | 0.014 | 0.886 | 0.376 |
| Video games hours per day | -0.006 | -0.402 | 0.688 |
| Extracurricular Activities | -0.017 | -0.600 | 0.549 |
| Parental Support | -0.087 | -2.393 | 0.017 |
| Parental Control | -0.029 | -0.962 | 0.336 |
| Sensation Seeking wave 1 | 0.483 | 18.455 | 0.000 |
| Movie Reckless driving exposure | 0.466 | 1.476 | 0.140 |
| **Correlation: Reckless driving with inattentive driving** | 0.653 | 13.540 | 0.000 |
| **Correlation: Sped through yellow with run red light** | 1.310 | 4.809 | 0.000 |
| **Intercepts** |  |  |  |
| Sensation seeking wave 4 | 1.593 | 5.782 | 0.000 |
| **Thresholds** |  |  |  |
| Over Speed Limit | 3.432 | 2.056 | 0.040 |
| Tailgated | 5.781 | 5.026 | 0.000 |
| Weaved in and out of traffic | 5.161 | 5.382 | 0.000 |
| Cross double yellow line to pass | 4.513 | 7.114 | 0.000 |
| Sped through a yellow light | 2.713 | 2.607 | 0.009 |
| Failed to use seatbelt | 4.542 | 10.105 | 0.000 |
| Failed to yield | 2.142 | 1.577 | 0.115 |
| Ran red light | 1.626 | 2.600 | 0.009 |
| Ignored stop sign | 2.072 | 2.215 | 0.027 |
| **Residual Variances** |  |  |  |
| Sensation seeking wave 4 | 0.256 | 26.192 | 0.000 |
| Reckless driving | 1.000 |  |  |
| Inattentive driving | 1.000 |  |  |
